# TREM2 promotes Aβ phagocytosis by upregulating C/EBPα-dependent CD36 expression in microglia

**DOI:** 10.1038/s41598-017-11634-x

**Published:** 2017-09-11

**Authors:** Su-Man Kim, Bo-Ram Mun, Sun-Jun Lee, Yechan Joh, Hwa-Youn Lee, Kon-Young Ji, Ha-Rim Choi, Eun-Hee Lee, Eun-Mi Kim, Ji-Hye Jang, Hyeong-Woo Song, Inhee Mook-Jung, Won-Seok Choi, Hyung-Sik Kang

**Affiliations:** 1 School of Biological Sciences and Technology, Chonnam National University, 77 Yongbong-ro, Buk-gu, Gwangju 500-757 South Korea; 2 Medical Device Development Center, Daegu-Gyeongbuk Medical Innovation Foundation, Cheombok-ro 80, Dong-gu, Daegu, 701-310 South Korea; 3 Department of Nursing, Nambu University, 23 Chumdan Jungang-ro, Gwangsan-gu, Gwangju, 506-706 South Korea; 4 Research Division for Biotechnology, Advanced Radiation Technology Institute (ARTI), Korea Atomic Energy Insitute (KAERI), 29 Geumgu-gil, Jeongeup-si, Jeollabuk-do 580-185 South Korea; 5 Department of Biochemistry and Biomedical Sciences, College of Medicine, Seoul National University, Seoul, Republic of Korea; 6 Predictive Model Research Center, Korea Institute of Toxicology, 141 Gajeong-ro, Yuseoung-gu, Daejeon, 34114 Republic of Korea

## Abstract

TREM2 plays a critical role in the alleviation of Alzheimer’s disease by promoting Aβ phagocytosis by microglia, but the detailed molecular mechanism underlying TREM2-induced direct phagocytic activity of Aβ remains to be revealed. We found that learning and memory functions were improved in aged TREM2 TG mice, with the opposite effects in KO mice. The amount of phagocytosed Aβ was significantly reduced in the primary microglia of KO mice. CD36 expression in primary microglia was greater in TG than in WT mice but was substantially decreased in KO mice. The expression of C/EBPα, an upstream transcriptional activator of CD36, was also elevated in primary microglia of TG mice but decreased in KO mice. The transcription of CD36 was markedly increased by TREM2 overexpression, and this effect was suppressed by a mutation of the C/EBPα binding site on the CD36 promoter. The TREM2-induced expression of CD36 and C/EBPα was inhibited by treatment with PI3K/AKT signaling blockers, and phosphorylation of AKT was elevated in TREM2-overexpressing BV2 cells. The present study provides evidence that TREM2 is required for preventing loss of memory and learning in Alzheimer’s disease by regulating C/EBPα-dependent CD36 expression and the consequent Aβ phagocytosis.

## Introduction

Alzheimer’s disease (AD), one of the major neurodegenerative disorders, is characterized by the aggregation of amyloid-beta (Aβ)^1^. The failure of Aβ clearance or the disruption of Aβ homeostasis results in the accumulation of Aβ in the brain^2^. Extracellular Aβ deposition, known as Aβ plaques, causes neuronal loss and cognitive decline^3^. Soluble or small oligomeric forms of Aβ have also been postulated to have deleterious effects in the brain and to promote the progression of AD by inducing changes in synaptic function, behavioral deficits and neuronal degeneration^4, 5^.

Microglia are immune cells that contribute to phagocytosis in the central nervous system, and they play critical roles in the uptake of Aβ as well as of dead neural cells. The microglia-mediated phagocytosis of Aβ and apoptotic cellular substances is mainly accomplished by cell surface receptors, such as scavenger receptors and triggering receptor expressed on myeloid cells 2 (TREM2)^6^. CD36, a member of the class B scavenger receptor family, has previously been shown to transport various molecules, such as fatty acids, collagen and oxidized low-density lipoproteins, into cells^7, 8^. Previous studies have revealed that CD36 binds to Aβ as a major pattern-recognition receptor expressed on microglia^9^ and that impaired recycling of CD36 reduces the phagocytic efficiency of microglia toward Aβ^10^. Although transcriptional activation of CD36 has been shown to be induced by overexpression of the transcription factor C/EBPα in adipocytes, myoblasts and macrophages^11^, the precise mechanisms involved remain unclear.

TREM2 is expressed on osteoclasts, dendritic cells, macrophages and microglia^12–14^. Signaling through TREM2 is required for its association with DAP12, which contains an immune-receptor tyrosine based activation motif (ITAM)^15^. Phosphorylation of the ITAM activates the tyrosine kinase Syk, which initiates downstream intracellular signaling through multiple mediators, such as PI3K, AKT and ERK^16^. Mutation of either TREM2 or DAP12 results in defective phagocytosis and the development of progressive dementias, such as Nasu-Hakola disease (NHD), frontotemporal dementia (FTD) and AD^17, 18^. Remarkably, the mutation of TREM2 impairs its binding to anionic lipids, which are accumulated during Aβ deposition and neuronal loss, thereby attenuating microglial detection of damage-associated lipids^19, 20^. TREM2, therefore, stimulates microglial survival as well as phagocytosis of Aβ, and its mutation has been identified as a risk factor for AD. To date, however, the regulatory mechanisms underlying TREM2 signaling in Aβ phagocytosis or AD pathogenesis have not been delineated.

Our previous study demonstrated that expression of C/EBPα and CD36 is markedly increased in the adipose tissues of TREM2 TG mice compared with WT mice^21^. In addition, CD36 expression is upregulated by C/EBPα in adipocytes, myoblasts and macrophages^11^. Taken together, these findings allow us to evaluate whether TREM2 is capable of promoting phagocytic activity of microglia via the regulation of C/EBPα and CD36 expression and to assess the effect of TREM2 on the behavior of aged TG and KO mice. Our present observations provide the first evidence that TREM2 increases CD36 expression by upregulating C/EBPα, thereby enhancing Aβ phagocytosis by microglia and preventing the loss of learning and memory with aging.

## Results

### TREM2 TG mice showed enhanced learning and memory, whereas a defective tendency was observed in KO mice

**Figure 1:**
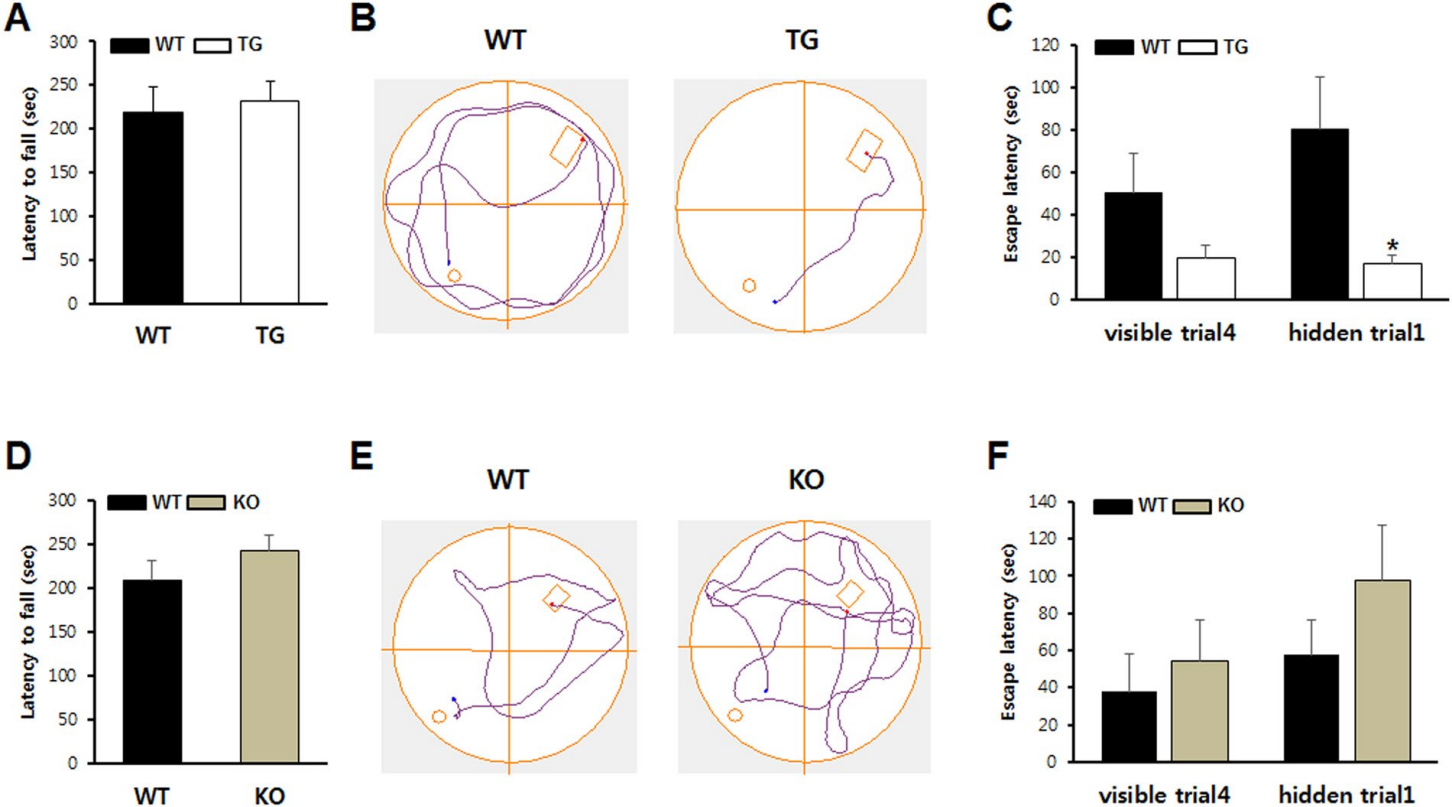
Learning and memory was enhanced in TREM2 TG mice, but TREM2 KO mice showed a tendency toward defects. (**A**) Motor behavior was analyzed using the rotarod test in TREM2 TG mice (n = 8) and WT littermates (n = 8) as described in the Methods section. (**B**) Representative traces of swimming plots in the Morris water maze test of TREM2 TG and WT littermates. **(C)** Bar graphs calculated from data obtained from the Morris water maze test. Visible trial 4 is the last visible platform trial on day 1 before testing, representing the baseline. During testing on day 2, the time spent by each mouse before arriving at the hidden platform during trial 1 (hidden trial 1) was measured as the escape latency. (**D**) Rotarod test in TREM2 KO (n = 6) and WT littermates (n = 7). (**E**) Plots of swimming traces in TREM2 KO and WT littermates. **(F)** Bar graphs calculated from data obtained from the Morris water maze test. Data are presented as the mean ± SEM. One-way analysis of variance with Tukey’s post hoc multiple comparisons test was performed for all statistical analyses (*p < 0.05; other comparisons were not significant).

The basal locomotor activity did not differ between TREM2 Transgenic (TG) and wild-type (WT) mice as confirmed using the rotarod test (Fig. 1A). To test the effect of TREM2 on spatial memory, we performed the Morris water maze test. There was no significant difference in swimming speed between TREM2 TG and WT mice, confirming that overexpression of TREM2 does not affect basal locomotion (see Supplementary Fig. S1A). Interestingly, TREM2 TG mice showed a significant enhancement in spatial memory. An indicator of learning impairment, the ratio of the latency on the first trial of day 2 to that on the last trial of day 1 (D2T1/D1V4), was significantly decreased in TREM2 TG mice (Fig. 1B,C). TREM2 KO and WT mice showed no differences in motor deficit in the rotarod test (Fig. 1D) or in the swimming speed in the Morris water maze test (see Supplementary Fig. S1B). However, TREM2 KO mice showed a tendency towards defective learning and memory compared to WT mice in novel object recognition (see Supplementary Fig. S2) and in the Morris water maze test, although the differences were not significant in the Morris water maze test (Fig. 1E,F). These results indicate that TREM2 expression levels in the brain are positively correlated with learning and memory function.

### Aβ protein levels were increased in the brain of TREM2 KO mice

To verify the expression pattern, TREM2 was detected in the brains of TG mice by real-time PCR, flow cytometry and immunofluorescence staining. TREM2 mRNA expression was ubiquitously increased by CMV promoter-driven TREM2 overexpression in all brain cells (see Supplementary Fig. S3A). However, the TREM2 expression level was much higher in CD11b-positive microglia of TG mice than CD11b-negative cells (see Supplementary Fig. S3A). TREM2 protein expression was also detected in CD11b-negative cells (see Supplementary Fig. S3B), such as astrocytes (GFAP-positive) and neuron cells (NeuN-positive) of TG mice (see Supplementary Fig. S3C). Although TREM2 was ubiquitously overexpressed in the brain cells of TG mice, the effects of TREM2 in the brain of TG mice would be due to microglia, considering that DAP12 essential for TREM2 signaling is expressed only in microglia^22^.

Next, to determine which cell types in the central nervous system express the TREM2 transgene, we also measured TREM2 expression in E14 and neonatal cultures by western blot and flow cytometric analysis. Western blotting showed greater TREM2 expression in neonatal cultures from TG mice than in those from WT mice, but TREM2 was not detectable in E14 cultures from either genotype (see Supplementary Fig. S4A). Similar expression levels of TREM2 are shown in the dot plot and histogram from flow cytometric analysis using a CD11b antibody specific for microglia (see Supplementary Fig. S4B). These data indicated that in both TG and WT mice, TREM2 was highly expressed in microglia.

**Figure 2:**
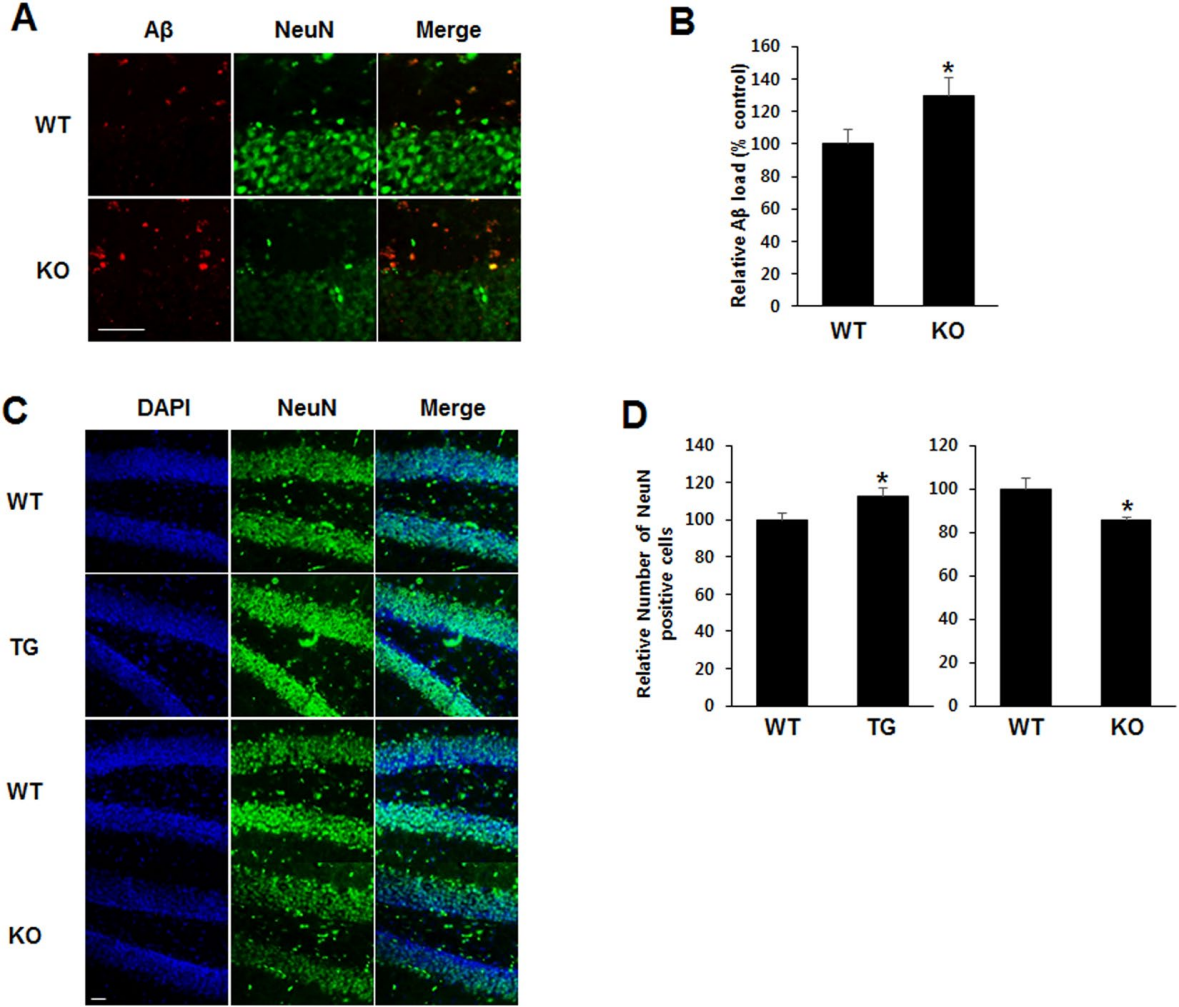
Aβ protein levels were increased in KO mice. (**A**) Immunohistochemistry for the neuronal marker NeuN and for Aβ was performed in hippocampal slices from WT and TREM2 KO mice (24 month-old). Scale Bar: 50 μm **(B)** Relative Aβ load in the hippocampus, DG was represented as a bar graph. **(C)** Immunostaining shows that the TREM2 expression level could affect neuronal cell number in WT, TREM2 TG and TREM2 KO mice. Scale Bar: 10 μm **(D)** Number of NeuN-positive cells in hippocampus, DG. Data are presented as the mean ± SEM (*p < 0.05, Student’s t test).

It has been previously reported that levels of Aβ protein deposition in the brain were associated with impairment of learning and memory^23, 24^. Based on these previous reports, the altered learning and memory in TG and KO mice, shown in Fig. 1, may be caused by TREM2-induced reductions and increases in Aβ protein expression in these mice. Thus, we investigated the protein levels of endogenous murine Aβ in the brain of KO mice. The endogenous Aβ protein levels as determined by immunofluorescence staining were elevated in the brain of KO mice compared with those from WT controls (Fig. 2A). After densitometric quantitation from immunofluorescence staining, the expression levels of Aβ protein were calculated as percentage and shown in bar graphs (Fig. 2B). Compared with WT brains, the numbers of NeuN-positive cells were higher in TG and lower in KO mice (Fig. 2C). The relative number of NeuN-positive cells was quantified and are shown as bar graphs (Fig. 2D). These results suggested that Aβ accumulation might be prevented by TREM2, which consequently increased the number of neurons in the brain.

### The protective effect of TREM2 against Aβ-induced neurotoxicity

**Figure 3:**
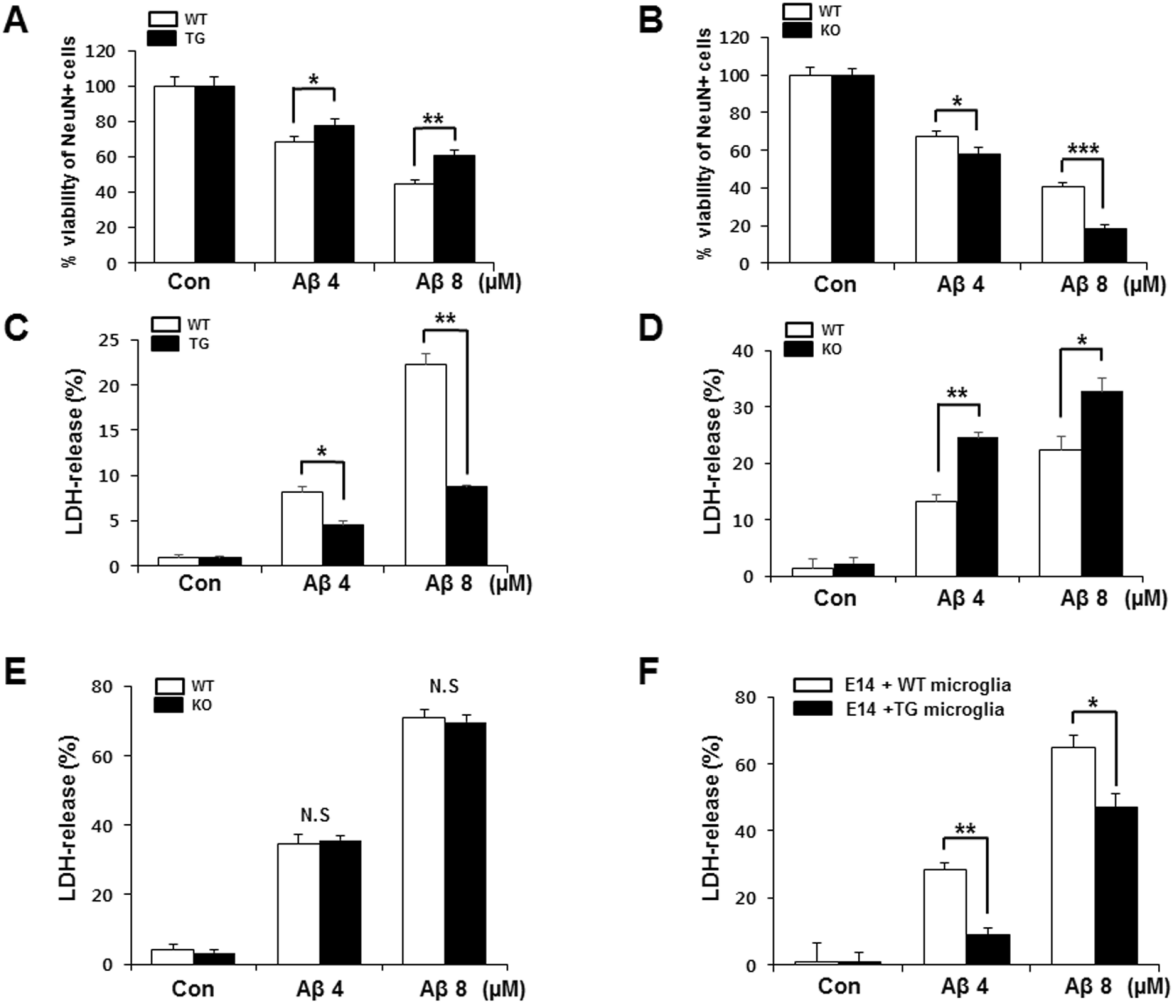
TREM2 increased the viability of neuronal cells in the presence of Aβ. Neonatal and embryonic day 14 (E14) neuronal cells isolated from the brains of mice treated with Aβ for 48 h. **(A)** Neuronal cells were stained with anti-NeuN and Alexa 488-conjugated secondary antibody. The number of NeuN-positive cells was quantified and calculated as the percentage of viable cells. **(B)** The percentage of NeuN-positive cells. **(C)** Amount of released LDH in the supernatant of neonatal cells treated with Aβ. **(D)** The percentage of released LDH. **(E)** The percentage of released LDH in KO and WT E14 cells treated with Aβ. **(F)** Amount of released LDH in the supernatant of TG or WT microglia co-cultured with E14 cells in the presence of Aβ. Data shown are representative of five independent experiments, and the error bars represent the mean ± SEM of five independent experiments. (*p < 0.05; **p < 0.01; ***p < 0.005; N.S.: not significant).

Aβ oligomers (Aβ_1-42_) are well known to induce neurotoxicity and to decrease neuronal viability^25^. To investigate the effect of TREM2 on neuronal cell damage, isolated neonatal neural cells, which also contain non-neuronal cells, from TG, KO and WT mice were treated with Aβ oligomers. The production of Aβ oligomers was confirmed by western blot (see Supplementary Fig. S5). After treatment, the numbers of remaining neurons were measured using immunofluorescence analysis with a NeuN (neuron-specific) antibody. Cytotoxicity was measured by the lactate dehydrogenase (LDH) release assay. In all examined neonatal neurons from TG, KO and WT mice, the frequency of NeuN-positive cells was dose-dependently decreased by treatment with Aβ oligomers. As shown in bar graphs calculated from immunofluorescence data, the numbers of NeuN-positive cells treated with Aβ oligomers at 4 μM and 8 μM were increased by 9% and 16% in TG mice compared with WT mice, respectively (4 μM: 68 ± 4% in WT *vs*. 77 ± 3% in TG, 8 μM: 44 ± 4% in WT *vs*. 60 ± 3% in TG) (Fig. 3A). However, the numbers of neurons were decreased by 10% and 17% in KO mice (4 μM: 67 ± 7% in WT *vs*. 57 ± 6% in KO, 8 μM: 40 ± 8% in WT *vs*. 17 ± 7% in KO) (Fig. 3B), suggesting that the neonatal neurons of TG mice were less sensitive to Aβ-induced neuronal toxicity than were neonatal neurons from WT mice. In another cell viability test using the LDH release assay, LDH levels in the culture supernatant (Sup) of neonatal neurons were lower in TG mice than in WT mice (4 μM: 8.20 ± 0.49% in WT *vs*. 4.53 ± 0.47% in TG, 8 μM: 22.23 ± 1.26% in WT *vs*. 8.73 ± 0.12% in TG) (Fig. 3C), and much higher levels of LDH release were observed in KO mice (4 μM: 13.29 ± 1.16% in WT *vs*. 24.53 ± 1.01% in KO, 8 μM: 22.29 ± 2.44% in WT *vs*. 32.82 ± 02.26% in KO) (Fig. 3D). To distinguish viable from non-viable cells in the immunofluorescent staining and LDH assays, flow cytometry was performed on neonatal cultured cells treated with Aβ. The neonatal cultured cells included approximately 30% NeuN-positive cells and 70% NeuN-negative cells. The fraction of propidium iodide (PI)-positive neuronal cells was increased by Aβ treatment. However, the fraction of PI-positive cells among the NeuN-negative cells was not changed by Aβ treatment (see Supplementary Fig. S6). These data suggested that our experiments can be used for measuring the viability of neurons. Intriguingly, the viability of embryonic day 14 (E14) neuronal cells treated with Aβ oligomers from TG and WT mice showed no differences in the LDH assay (Fig. 3E). However, after treatment with Aβ oligomers, the co-culture of E14 neuronal cells and primary microglia from TG mice showed significantly greater cell viability than cultures from WT mice (Fig. 3F). These data suggested that the lack of difference in the viability of neuronal cells between TG and WT mice might be due to an absence of microglia at E14 and that TREM2 on microglia might play an essential role in protecting neuronal cells against Aβ-induced cellular toxicity.

### TREM2 is required for the direct clearance of Aβ by microglia

**Figure 4:**
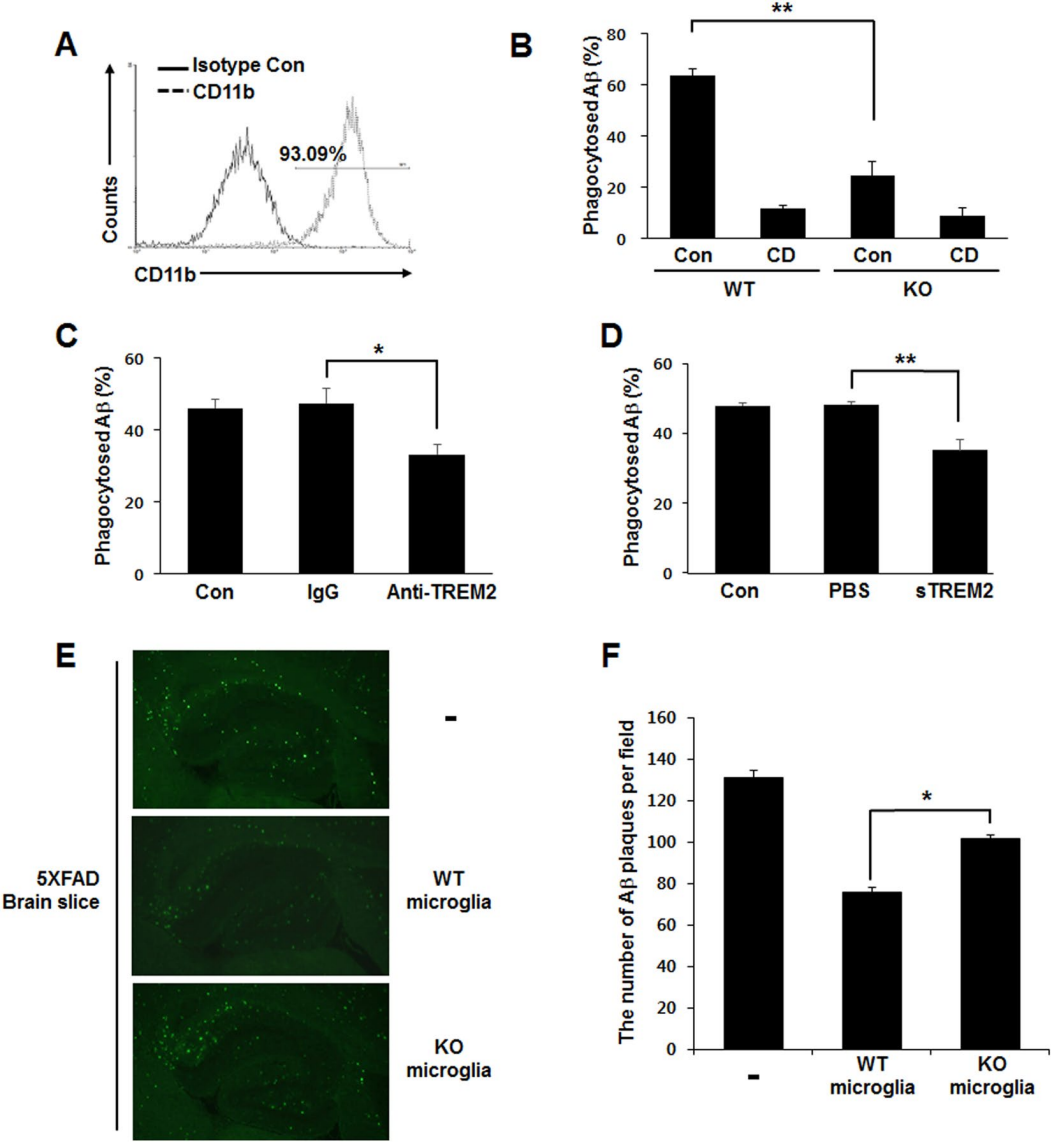
Deficiency of TREM2 signaling impairs the phagocytic activity of microglia. (**A**) Primary microglia of WT or KO mice were stained with PE-conjugated anti-CD11b or isotype control antibody, and the purity of primary microglia was determined using flow cytometric analysis. **(B)** Primary microglia were treated with 8 μM Aβ in the presence of 10 μM cytochalasin D (CD) as an inhibitor of actin-mediated phagocytosis, and the percentage of Aβ phagocytosed was determined using the phagocytosis assay. **(C)** The percentage of Aβ phagocytosed by BV2 cells after treatment with anti-TREM2 (1 μg/ml). **(D)** The percentage of Aβ phagocytosed by BV2 cells after treatment of soluble TREM2 (sTREM2, 2 μg/ml). **(E**) Representative fluorescence microscopic views (20×) from brain slices of 5XFAD mice stained with Thioflavin T after co-culture with primary microglia. Scale bar: 200 μm **(F**) The numbers of Aβ plaques quantified from the brain slice assay in (**E**). Data shown are representative of three independent experiments, and the error bars indicate the mean ± SEM of three independent experiments. (*p < 0.05; **p < 0.01).

Although TREM2 has been reported as a phagocytic receptor on microglia^18, 26, 27^, little is known regarding the molecular mechanism of Aβ phagocytosis regulated directly by microglial TREM2. To determine how and whether Aβ phagocytosis is directly regulated by microglial TREM2, phagocytic efficiency was evaluated by measuring the amounts of phagocytosed fluorescent beads, Aβ oligomers and plaques in primary microglia purified from WT and KO mice. The purity of primary microglia was greater than 93% as determined by flow cytometry (Fig. 4A). The amount of phagocytosed Aβ assessed by ELISA was decreased by approximately 40% in primary microglia of KO mice compared with WT mice (63.67 ± 2.73 in WT *vs*. 24.67 ± 5.38 in KO), with no difference when treated with cytochalasin D (CD), a phagocytosis inhibitor (Fig. 4B). To further confirm the phagocytic efficiency in primary microglia of KO mice, BV2 microglia cells were treated with anti-TREM2 antibody and soluble TREM2 (sTREM2) to block signaling through TREM2, and phagocytosed Aβ was then analyzed. Treatment with anti-TREM2 antibody or sTREM2 significantly reduced the amount of phagocytosed Aβ (Fig. 4D). In an *ex vivo* brain slice assay, the remaining Aβ plaques were measured by Thioflavin T staining after the addition of primary microglial cells from WT or KO mice to Aβ plaque-depositing brain slices of 5XFAD mice. The frequency of Aβ plaques was much higher in brain slices with primary microglia from KO mice than in those with microglia from WT mice (Fig. 4E). Figure 4F shows a bar graph of the quantification obtained from Thioflavin T staining. Furthermore, compared to WT mice, the number of primary processes was increased in TG but decreased in KO mice *in vivo* (see Supplementary Fig. S7). These data indicate that TREM2 on microglia is required for the direct clearance of Aβ in the brain.

### TREM2 enhanced the phagocytic efficiency of Aβ by inducing CD36 expression

**Figure 5:**
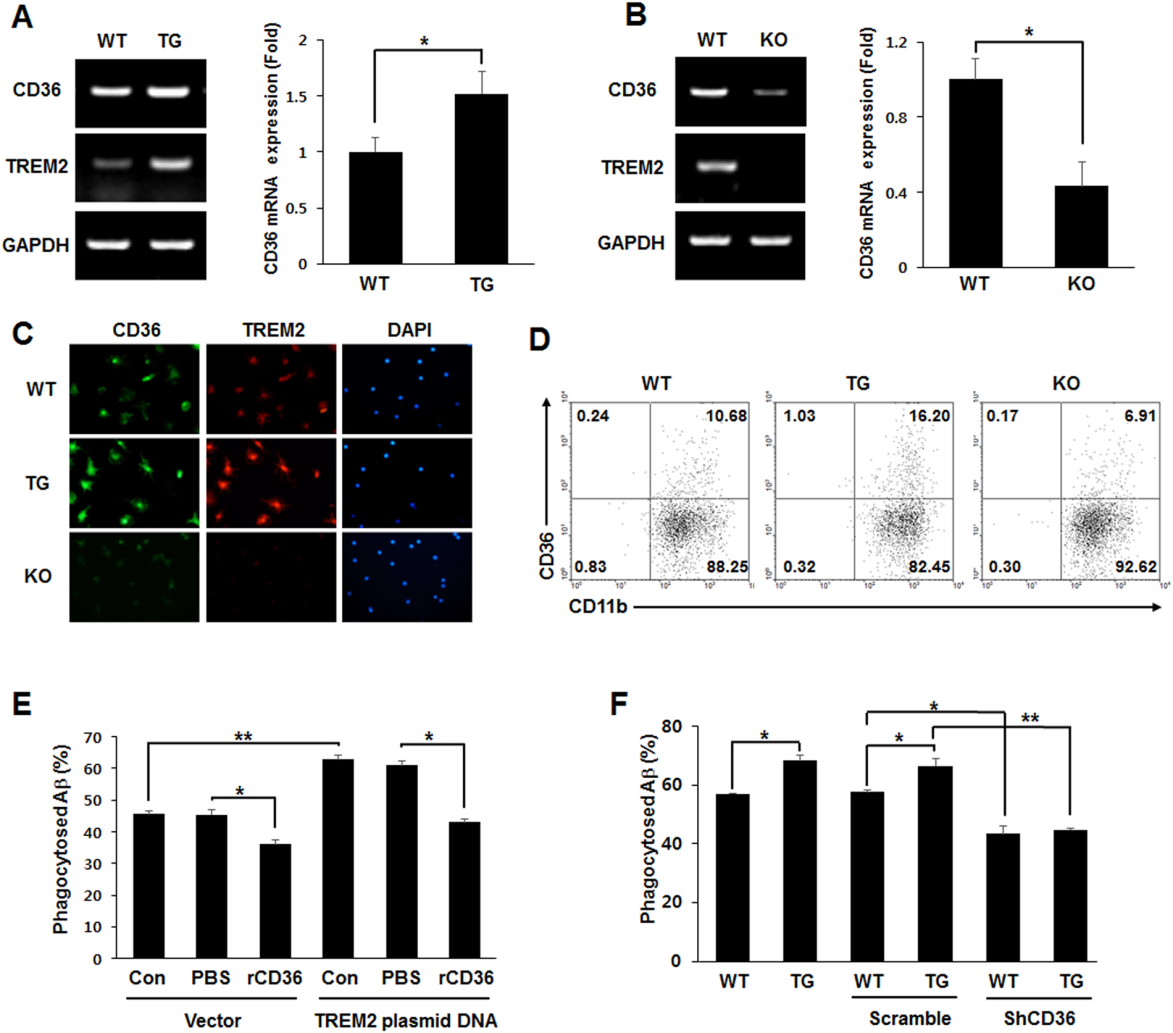
TREM2 promotes Aβ phagocytosis by upregulating CD36 in microglia. (**A**,**B**) CD36 mRNA expression was determined by semi-quantitative RT-PCR from primary microglia (left), and the relative mRNA expression levels were quantitatively analyzed by normalization against GAPDH (right panels) (full-length blots/gels are presented in Supplementary Fig. S8). **(C)** Immunofluorescent staining of primary microglia. **(D)** Surface expression of CD36 on primary microglia analyzed using flow cytometry. **(E)** BV2 cells were treated with recombinant CD36 (rCD36, 1 μg/ml) in the presence of Aβ (8 μM) at 24 h after transfection with a control vector or a plasmid encoding TREM2, and the phagocytosis assay was performed. **(F)** The percentage of phagocytosed Aβ in primary microglia infected with lentivirus encoding shRNA targeting CD36. Data shown are representative of three independent experiments, and the error bars indicate the mean ± SEM of three independent experiments. (*p < 0.05; **p < 0.01).

It has been reported that CD36, as a pattern recognition receptor on macrophages, mediates the clearance of Aβ as well as of microbial pathogens^9^. In addition, CD36 facilitates the receptor-mediated endocytosis of low-density lipoproteins in adipocytes^8^. Given that the expression of CD36 was upregulated by TREM2 during adipocyte differentiation in our previous study^21^, we investigated whether TREM2 could enhance Aβ phagocytosis by regulating CD36 expression in microglia. CD36 mRNA levels were upregulated in the primary microglia of TG mice (1.16 ± 0.14 in WT *vs*. 1.76 ± 0.19 in TG) (Fig. 5A), but they were markedly reduced in KO mice (3.85 ± 0.43 in WT *vs*. 1.66 ± 0.49 in KO) (Fig. 5B). Furthermore, compared with WT mice, CD36 protein expression in the primary microglia was increased in TG mice but decreased in KO mice, as determined by immunofluorescence staining (Fig. 5C). According to flow cytometric analysis, the population of CD11b^+^CD36^+^ primary microglia was higher in TG mice than in WT mice but was lower in KO mice (Fig. 5D). To confirm the phagocytosis of Aβ by TREM2-induced expression of CD36, the phagocytosis assay was performed in TREM2-overexpressing BV2 cells treated with recombinant CD36 (rCD36) as a binding competitor for CD36 on the cell surface membrane. The percentage of phagocytosed Aβ was also significantly enhanced in TREM2-overexpressing BV2 cells but reduced by treatment with rCD36 (60.93 ± 1.60 in TREM2-PBS *vs*. 43.08 ± 1.01 in TREM2-rCD36) (Fig. 5E). The enhanced percentage of phagocytosed Aβ was also observed in the primary microglia of TG mice compared with WT mice, and this was suppressed by the transduction of lentivirus encoding CD36 shRNA (shCD36) (57.06 ± 0.12 in WT *vs*. 68.23 ± 1.78 in TG, 57.6 ± 0.86 in WT-scramble *vs*. 66.59 ± 2.4 in TG-scramble, 43.3 ± 2.7 in WT-shCD36, 44.82 ± 0.58 in TG-shCD36) (Fig. 5F). These data suggest that TREM2 promotes Aβ phagocytosis by upregulating CD36 in microglia.

### TREM2 increases the expression level of CD36 by **upregulating C/EBP**α in microglia

**Figure 6:**
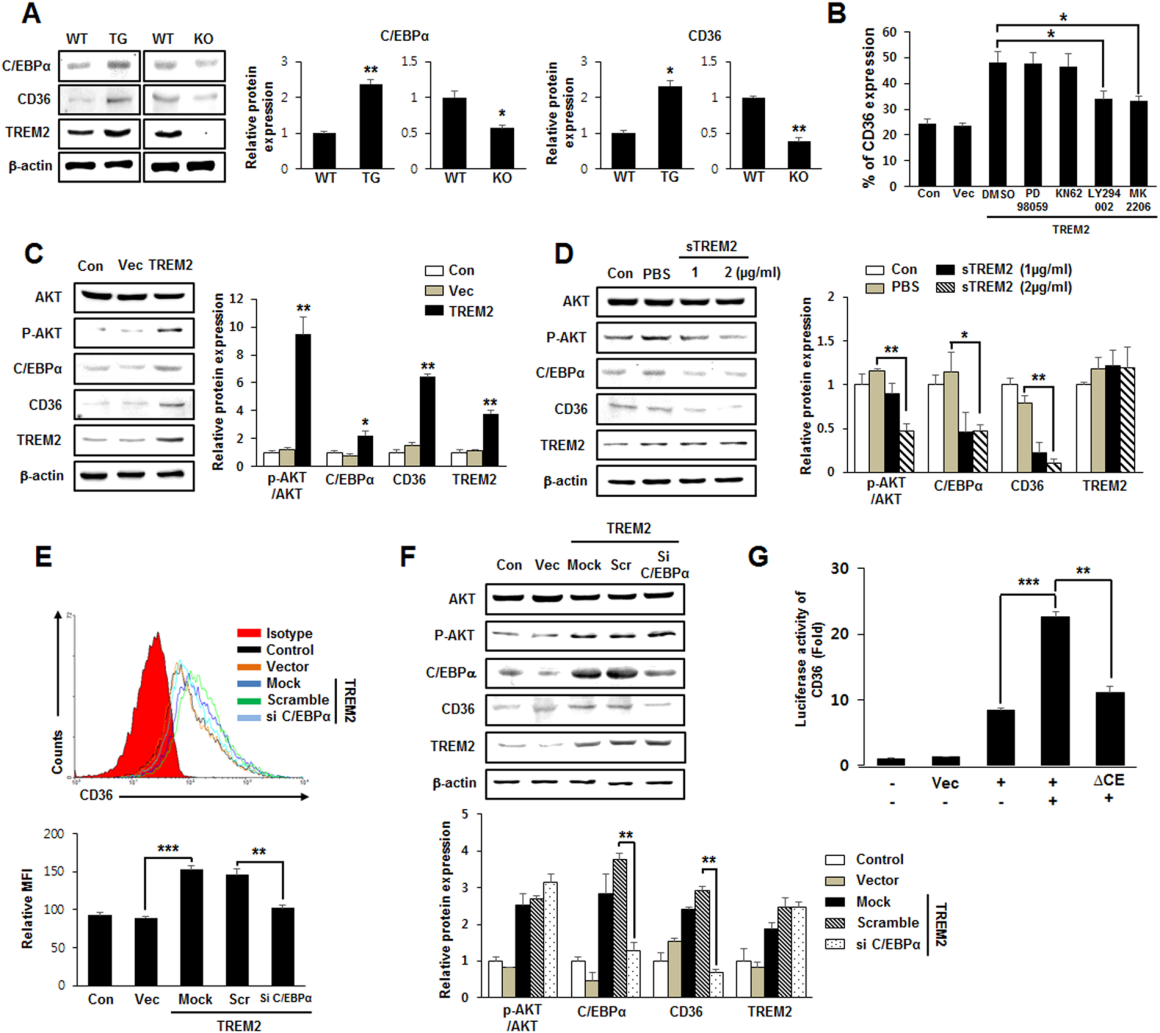
TREM2-induced C/EBPα promotes the transcription of CD36 in microglia. (**A**) The expression levels of C/EBPα and CD36 in primary mouse microglia were analyzed by western blot (full-length blots/gels are presented in Supplementary Fig. S9). **(B**) After transfection of BV2 cells with an empty vector or a plasmid encoding TREM2, the cells were treated for 24 h with signaling blockers, such as PD (PD98059), KN (KN62), LY (LY294002) and MK (MK2206), and cell surface expression of CD36 was analyzed using flow cytometry. The CD36-positive populations from data obtained from flow cytometry are represented as percentages in bar graphs. **(C)** Western blot analysis in BV2 cells transfected with vector or TREM2 (full-length blots/gels are presented in Supplementary Fig. S10). **(D)** Western blot analysis in BV2 cells treated with PBS or soluble TREM2 (sTREM2) (full-length blots/gels are presented in Supplementary Fig. S11). **(E)** After transfection of BV2 cells with vector or TREM2 and with scramble or C/EBPα siRNA, cell surface expression of CD36 was plotted as the mean fluorescence intensity (MFI) on a histogram of flow cytometric analysis. **(F)** Western blot analysis in BV2 cells transfected with the indicated expression or siRNA plasmids (full-length blots/gels are presented in Supplementary Fig. S12). The western blot and flow cytometry were quantified by using ImageJ or WinMDI software and are presented as bar graphs. **(G)** BV2 cells were co-transfected with the expression vector pGL3 basic-Luc, CD36-Luc or ΔCE-Luc and TREM2. After 48 h, luciferase activity was determined by a luminometer, and the data were normalized against β-galactosidase. All data shown are representative of three independent experiments, and the error bars indicate the mean ± SEM of three independent experiments. (*p < 0.05**p < 0.01; ***p < 0.001).

Although CD36 has been known to be highly expressed in macrophages and adipocytes^28^, and its expression is regulated by transcriptional activation of CCAAT/enhancer-binding protein alpha (C/EBPα) during adipocyte differentiation^11^, the TREM2-mediated, C/EBPα-dependent expression of CD36 has not yet been investigated. Western blots showed that compared with WT mice, the protein levels of C/EBPα in primary microglia were increased TG mice but decreased in KO mice, which correlated with results obtained for CD36 (Fig. 6A). To identify which signaling pathway is involved in TREM2-induced expression of CD36, CD36 expression was analyzed using flow cytometry in TREM2-overexpressing BV2 cells treated with signaling blockers for Erk (PD98059), calmodulin (KN62), PI3K (LY294002) and AKT (MK2206). The percentages of CD36-positive cells were increased by TREM2 overexpression and were significantly reduced by treatment with LY294002 or MK2206 but not by treatment with PD98059 or KN62 (Fig. 6B), suggesting that TREM2 may upregulate CD36 expression *via* the PI3K/AKT signaling pathway. Indeed, elevated levels of AKT phosphorylation were observed in TREM2-overexpressing BV2 cells compared with vector controls (Fig. 6C). However, blockade of TREM2 signaling by treatment of BV2 cells with sTREM2 resulted in decreased protein levels of both C/EBPα and CD36 (Fig. 6D). Flow cytometric analysis further revealed that the cell-surface expression levels of CD36 were increased by TREM2 overexpression but decreased by silencing C/EBPα expression in BV2 cells (Fig. 6E). The expression pattern of CD36 was confirmed by western blot analysis in C/EBPα-silenced BV2 cells (Fig. 6F). To further determine whether TREM2-induced C/EBPα can regulate the transcription of CD36 via its promoter, the luciferase activity of CD36 was evaluated in BV cells transfected with CD36-Luc or a mutant of C/EBPα binding element-Luc (ΔCE) and/or TREM2. The luciferase activity of CD36 was markedly increased by TREM2 overexpression, but it was significantly reduced in BV2 cells transfected with the ΔCE mutant (Fig. 6G). Furthermore, compared with WT mice, hippocampal CD36 protein expression was also increased in TG mice but was decreased in KO mice, as determined by immunofluorescence staining *in vivo* (see Supplementary Fig. S13). The expression of CD36 was elevated in TREM2- and Iba1-positive cells of TG compared with WT mice (see Supplementary Fig. S14). Taken together, these data suggest that TREM2-induced C/EBPα promotes the transcription of CD36 in microglia.

## Discussion

Here, we elucidated the molecular mechanism by which TREM2 promotes the transcription of CD36 by upregulating C/EBPα *via* the PI3K/AKT/CREB pathway in microglia. TREM2-induced upregulation of CD36 subsequently enhanced the phagocytosis of Aβ and protected neuronal cells against Aβ-induced cellular toxicity. In addition, TREM2-mediated Aβ phagocytosis was substantially impaired by the silencing of CD36 or C/EBPα. Furthermore, compared to WT mice, aged TG mice showed enhanced memory and learning with increased number of neurons, and contrasting results were observed in KO mice. Thus, the present study provides evidence that TREM2 is required for preventing the aging-related loss of memory and learning by regulating C/EBPα-dependent CD36 expression and consequently Aβ clearance.

Aging is one of the major risk factors for the development of various diseases, including AD, and the aged mouse model is useful for studying the early stage of Aβ pathophysiology^29^. Our previous study demonstrated that compared to WT mice, body weight is substantially increased in aged (15-month-old) TG mice fed a standard diet, indicating that TREM2 overexpression promotes age-related obesity^21^. In this study, 12-month-old aged TG and KO mice were used for behavioral tests and biochemical assays due to the sluggish movement caused by obesity in TG mice aged over 12 months. Although the aged TG and KO mice were not able to fully reflect the pathophysiology of AD, they can be used for functional studies of TREM2 in the regulation of Aβ homeostasis and the early pathogenesis of AD.

A recent study reported that within 3 days, the *in vitro* survival rate of TREM2 KO microglia is comparable with that of WT microglia, but at over 3 days in culture, it is lower than that of WT microglia^20^. Given that our *in vitro* and *ex vivo* phagocytosis assays were performed within 3 days, differences between genotypes in our phagocytosis data should not be due to differences in survival or the total number of microglia between TREM2 TG or KO mice and WT mice. Furthermore, TREM2 deficiency resulted in impaired phagocytosis of Aβ by reducing the survival and migration of microglia, indicating that TREM2 cannot directly regulate phagocytic activity^20^. Our findings in this study provide strong evidence that TREM2 directly enhanced the Aβ phagocytic activity of microglia, which was specifically abolished by blockade of TREM2. Furthermore, we also demonstrated that TREM2 could alleviate the Aβ oligomer-induced damage in neonatal neuronal cells but not in E14 neuronal cells due to the intrinsic absence of microglia. Thus, TREM2 may play a protective role against Aβ-induced neuronal toxicity, and this role is dependent on the presence of microglia.

Here, we demonstrate that TREM2 enhanced the function of learning and memory in the brain by preventing the accumulation of Aβ during aging, thereby increasing the viability of neuronal cells by protecting them from Aβ-induced cellular toxicity. The prevention of Aβ accumulation was accomplished by promoting TREM2-mediated phagocytic activity due to the activation of C/EBPα and subsequent upregulation of CD36 in microglia. Our findings suggest that TREM2 may be required for Aβ clearance at the onset of early-stage AD. Thus, TREM2 may play an indispensable role in the regulation of Aβ homeostasis and provide a useful strategy for the prevention and therapy of AD.

## Methods

### Generation of TREM2 Transgenic Mice

Pronuclear microinjection was performed to generate transgenic (TG) mice overexpressing TREM2 as described previously^30^. Mouse TREM2 cDNA was amplified by RT-PCR using the forward primer 5′-GAATTCGC CCTTGGCTGGCTGCTGGCA-3′ and the reverse primer 5′- GTACGTGAGAGAATTC-3′. The PCR product was cloned into the pcDNA3.1 expression vector, which contains the CMV promoter (Invitrogen, Carlsbad, CA). A fertilized egg of a C57BL/6 mouse was microinjected with the pure isolated recombinant TREM2 gene according to a standard protocol (Macrogen, Seoul, Republic of Korea). The integration of the TREM2 transgene was validated by Southern blotting. TREM2 TG mice were maintained in a C57BL/6 genetic background and genotyped by RT-PCR prior to use in the experiments.

### Animals

TREM2 transgenic (TG), TREM2 knock out (KO) mice and wild-type (WT) littermates were created in the C57BL/6 background. 5XFAD mice were maintained on a mixed B6/SJL background. All mice were maintained under specific pathogen-free conditions, and any offspring were genotyped using RT-PCR prior to use in the experiments. Mice were individually housed and maintained on a 12 h light/dark cycle at 22 ± 1 °C. All experiments using mice were performed according to the guidelines of the Institutional Animal Care Committee of Chonnam National University. All animal experimental protocols were reviewed and approved by the Chonnam National University Institutional Animal Care and Use Committee (CNU IACUC-YB-2013-7).

### Culture of embryonic and neonatal neuronal cells and BV2 cells

Neonatal mixed culture was performed as previously described^31^ with some modifications. Briefly, the meninges were removed from the brains of E14 embryos or 2-day-old mouse pups, and the forebrains were minced in 5% FBS-PBS, dissociated, and then filtered (40-μm cell strainer; BD Biosciences, San Diego, CA). Embryonic and neonatal neuronal cells were seeded at 10^5^ cells on an Aclar coverslip coated with poly-D-lysine (Ted Pella Inc., Redding, CA) and cultured in DMEM (Gibco, Big Cabin, OK) supplemented with 10% FBS and 1% penicillin/streptomycin at 37 °C and 5% CO_2_ for 3 days prior to Aβ treatment. BV2 cells were cultured using the same culture conditions as the embryonic and neonatal neuronal cells.

### Culture of primary microglia

The forebrains from 2-day-old mouse pups were filtered through a cell strainer (40 μm; BD) after separation and dissociation in 5% FBS-PBS. Cells were seeded (4.5 × 10^6^ cells/75-mm flask) and incubated in 10% FBS-DMEM containing 5 ng/ml MCSF or GM-CSF (Sigma-Aldrich, St. Louis, MO). After 2 weeks in culture, the flasks were shaken for 5 h (125 rpm, 37 °C) to harvest detached microglia.

### Transfection of BV2 cells by electroporation and viral infection of primary microglia

BV2 cells were transiently transfected with pcDNA3.1 expression vectors containing the TREM2 gene or C/EBPα siRNA (Santa Cruz Biotechnology, Santa Cruz, CA) by electroporation using an Amaxa Nucleofector® (Lonza, Walkersville, MD), as described in the manufacturer’s protocol. Primary microglia were infected with 10^8^ infection units/ml of lentivirus containing shRNA plasmids targeting mouse CD36 (Abcam, Cambridge, MA) in the presence of polybrene (8 μg/ml) (Sigma).

### Aβ oligomerization and lactate dehydrogenase (LDH) release assay

Oligomerization of Aβ peptide (American Peptide Company, Sunnyvale, CA) was dissolved in HFIP (hexafluropropan-2-ol; Sigma–Aldrich, 4 °C) at 2.5 mg/ml. The peptide solution was incubated at 25 °C for 1 h, after which HFIP was removed using a Speed Vac (Thermo Savant). The resulting dried Aβ-(1–42) film was stored at −70 °C. DMSO (Sigma–Aldrich) was added to the tube containing dried Aβ-(1–42) peptide to yield a final concentration of 5 mM. The tube was immediately processed as follows. To obtain oligomers, PBS was immediately added to dissolve the peptide at a final concentration of 100 μM. The mixture was incubated at 4 °C for 24 h. The production of Aβ oligomers was confirmed by western blot (see Supplementary Fig. S7). Cells were incubated with Aβ oligomers for 48-72 h, and cell death and lysis were then evaluated by measuring the amount of released LDH using the cytoTox 96® Non-Radioactive Cytotoxicity Assay Kit (Promega, Madison, WI) according to the manufacturer’s instructions.

### Immunofluorescence staining

Neonatal neuronal cells treated with Aβ oligomers and primary microglia were fixed with 4% paraformaldehyde. The cells were incubated with primary antibodies for NeuN (1:1000; Millipore, Marlborough, MA, cat: MAB377), TREM2 (1:500; Abcam, cat: ab86491) and CD36 (1:500; Santa Cruz Biotechnology, cat: sc-7309) at room temperature for 24 h. After washing three times with PBS containing 0.1% Triton X-100 (Sigma), the cells were incubated with appropriate Alexa 488- or 560-conjugated secondary antibodies (1:2000; Thermo Fisher Scientific, Waltham, CA) for 1 h and counterstained with DAPI, followed by mounting with VectaShield mounting medium (Vector Laboratories, Burlingame, CA). Perfused and fixed mouse brains were sliced using a cryostat (Leica). Brain sections (30 μm thick) were blocked in 1 M PBST (PBS with 0.25% Triton X-100) containing 3% bovine serum albumin and 3% goat serum for 1 h at room temperature. The slices were incubated with primary antibodies at 4 °C for 16–24 h. The primary antibodies used were as follows: mouse anti-NeuN (1:100, Millipore), rabbit anti-Iba1 (1:500, Wako, cat: 019–19741), rabbit anti-Aβ (1:1000, Millipore, cat: ab5078p). Brain sections were incubated with Alexa Fluor 488- or 594-conjugated anti-mouse or anti-rabbit IgG (1:1000, Invitrogen). A Leica DM LB2 (Leica) with was used to capture images. The images of hippocampus were taken using a 20x objective. Boxes of same area were assigned over each region in dentate gyrus (DG) and NeuN-positive cells were quantitated in the selected region using ImageJ software (NIH). The number of NeuN positive cells was counted in 4 slices per genotype, using the cell counter plug-in of ImageJ to measure the areas with background subtraction in advance based on a threshold set. For Aβ quantification, the fluorescence level in the DG region was measured in 5 slices per genotype. The intensity of Iba1-positive cells was quantified using ImageJ. Average number of primary processes in each Iba-1 positive cell in the DG region was calculated in 5 slices per genotype.

### Semi-quantitative RT-PCR

The primer sequences for RT-PCR were as follows: TREM2, 5′-ATGGGACCTCTCCACCAGTT-3′ and 5′-GGGTCCAGTGAGGATCTGAA-3′; CD36, 5′- GCCAGTCGGAGACATGCTTA-3′ and 5′-ATTGAGTCCTGGGGCTCCTG-3′; and GAPDH, 5′-CATCACAACCACTCCCACTG-3′ and 5′-GATGGACCCCCGTCATAAGT-3′. All PCR amplifications were performed at 95 °C for 1 min, 56 °C for 1 min, and 72 °C for 2 min, with 27 cycles for GAPDH and 30 cycles for all other reactions. An additional extension step was performed at 72 °C for 10 min. PCR products were electrophoresed and visualized using ethidium bromide staining.

### Western blot and flow cytometric analysis

Cells were lysed in protein lysis buffer [20 mM HEPES (pH 7.9), 100 mM KCl, 300 mM NaCl, 10 mM EDTA, 0.5% Nonidet P-40, 1 mM Na_3_VO_4_, 1 mM PMSF, 10 µg/mL aprotinin, and 10 µg/mL leupeptin] for 30 min on ice. The protein concentrations were measured using Bradford reagent (Bio-Rad, Berkeley, CA). Cell lysates containing equal amounts of protein were subject to sodium dodecyl sulfate-polyacrylamide gel electrophoresis (SDS-PAGE) and transferred to a polyvinylidene fluoride (PVDF) membrane (Millipore) for immunoblotting. The membrane was incubated with blocking buffer (1% BSA and 5% skim milk in PBS) at 4 °C overnight and then washed three times with TBS-T [50 mM Tris (pH 7.4), 150 mM NaCl, 0.05% Tween 20]. The membrane was incubated with antibodies at 4 °C for 12 hours. Antibodies for western blot analysis were as follows: anti-CD36 (1:2000), anti-TREM2 (1:3000), anti-C/EBPα (1:1000, Santa Cruz Biotechnology, cat: sc-9314), anti-CREB (1:1000, Santa Cruz Biotechnology, cat: sc-186), anti-p-CREB (1:2000, Santa Cruz Biotechnology, cat: sc-7978), anti-β-actin (1:5000; Santa Cruz Biotechnology, cat: sc-47778), anti-AKT (cat: #9272s), and anti-p-AKT (1:2000; Cell Signaling Technology, cat: #9271s). For flow cytometric analysis, primary microglia and BV2 cells were treated with PD98059, KN62, LY294002 and MK2206 (Sigma). The cells were stained at 4 °C for 20 min with PE-conjugated anti-CD36 (1:500, cat: 562702) and FITC-anti-CD11b (1:1000; BD, cat: 557396), followed by analysis with a FACS Calibur (BD).

### Phagocytosis and *ex vivo* brain slice assay

Phagocytosis assays for was performed as previously described^10^. Primary microglia were pretreated with 10 μM cytochalasin D (Sigma-Aldrich) as a negative control for 30 min and then incubated with Aβ oligomers for 2 h. BV2 cells were pretreated with a neutralizing antibody targeting TREM2 (R&D Systems, cat: MAB17291) or with sTREM2 (Sino Biological Inc., Beijing, China) to block TREM2 signaling and then incubated with Aβ oligomers for 2 h. An *ex vivo* brain slice assay was performed as previously described^10^. Briefly, brain sections of 18-month-old 5XFAD TG mice were cultured with 2 × 10^5^ primary microglia in hybridoma serum-free media (H-SFM; Invitrogen) containing 1% FBS and 5 ng/ml GM-CSF (R&D Systems) for 60 h. The sections were stained with 1% Thioflavin T.

### Plasmid construction and promoter assay

The promoter region of CD36 and a site-directed mutant of the C/EBPα element (ΔCE) were cloned as previously described^11^. BV2 cells were transfected with the CD36 promoter containing pGL3 basic plasmids (Upstate Biotechnology, Lake Placid, NY). The luciferase activity was measured using the MicroLumat Plus LB96V luminometer (Berthold Technologies, Bad Wildbad, Germany).

### Rotarod and Morris water maze tests

Rotarod and Morris water maze tests were conducted with 12-month-old TREM2 TG, KO or WT littermates. Mice were placed on a rotating drum, and the latency to fall from the accelerating rotarod (B.S. Technolab INC., Seoul, Korea) was measured. The rotarod speed was 4 rpm for 1 min (30 min interval), and the speed accelerated from 4 to 40 rpm for 5 min (eight trials with 1-h inter-trial intervals). The Morris water maze test consisted of a round container (110 cm in diameter, 40 cm in depth) filled with water (22 ± 2 °C) to a depth of 30 cm. The escape platform was placed in the middle of the northeast quadrant, 1 cm below the water level. Mice were placed next to the wall of the container, facing southwest, for the acquisition phase. The latency to reach the platform with a visible cue was measured in four trial sessions during 1 day, and the mice were allowed to stay on the platform for at least 180 sec after each trial. One day after the acquisition phase, a probe trial was performed by removing the visual cue and placing the mice in the southwest quadrant. The latency to reach the platform was measured in three trials. The mice were tracked and analyzed using ANY-maze software (Stoelting, Wood Dale, USA) and a video camera.

### Statistical analysis

For statistical analysis of the behavior tests, one-way analysis of variance (ANOVA) with Tukey’s post hoc multiple comparisons test was performed for all analyses. For the other statistical analyses, p-values were calculated using two-way ANOVA or Student’s t-test. The data were considered statistically significant when p-values were <0.05.

## Electronic supplementary material

Supplementary information
