# Supplementary material for: TREM2 promotes Aβ phagocytosis by upregulating C/EBPα-dependent CD36 expression in microglia
Source: Sci Rep. 2017 Sep 11;7:11118. doi: 10.1038/s41598-017-11634-x (PMC5593901; doi:10.1038/s41598-017-11634-x)
Supplement: Supplementary file 1 — Supplementary information [file 41598_2017_11634_MOESM1_ESM.pdf]

Supplementary Information

TREM2 promotes Aβ phagocytosis by upregulating C/EBPα dependent CD36 expression in microglia

Su-Man Kim[1], Bo-Ram Mun[1], Sun-Jun Lee[1], Yechan Joh[1], Hwa-Youn Lee[2], Kon-Young Ji[1], Ha-Rim Choi[3], Eun-Hee Lee[4], Eun-Mi Kim[6], Ji-Hye Jang[1], Hyeong-Woo Song[1], Inhee Mook-Jung[5], Won-Seok Choi[1*], and Hyung-Sik Kang[1*]

[1]School of Biological Sciences and Technology, Chonnam National University, 77 Yongbong-ro, Buk-gu, Gwangju 500-757.

[2]Medical Device Development Center, Daegu-Gyeongbuk Medical Innovation Foundation, Cheombok-ro 80, Dong-gu, Daegu 701-310.

[3]Department of Nursing, Nambu University, 23 Chumdan Jungang-ro, Gwangsan-gu, Gwangju 506-706.

[4]Research Division for Biotechnology, Advanced Radiation Technology Institute (ARTI), Korea Atomic Energy Insitute (KAERI), 29 Geumgu-gil, Jeongeup-si, Jeollabuk-do 580-185.

[5]Department of Biochemistry and Biomedical Sciences, College of Medicine, Seoul National University, Seoul, Republic of Korea

[6]Predictive Model Research Center, Korea Institute of Toxicology, 141 Gajeong-ro, Yuseoung-gu, Daejeon, 34114 Republic of Korea

* These corresponding authors contributed equally to this work.

Corresponding author information:

Dr. Hyung-Sik Kang, E-mail: kanghs@jnu.ac.kr; Tel.:(82)-062-530-2195; Fax: (82)-062-530-0315

Dr. Won-Seok Choi, E-mail: choiw@jnu.ac.kr; Tel.:(82)-062-530-1912; Fax: (82)-062-530-1912

Supplemental Experimental Procedures for novel object recognition test (Figure S2)

Novel object recognition test was performed as described (Su-Yeon Choi et al., Mice lacking the synaptic adhesion molecule Neph2/Kirrel3 display moderate hyperactivity and defective novel object preference. *Front. Cell. Neurosci.,* 28 July 2015). In the open field arena, mice were habituated for 20 min, 1 day before the test. Mice were placed in the arena to explore two objects for 10 min during the sample phase. Exploration time was measured for each object using anymaze software. In test phase, mice were replaced in the arena with one original object and a new one, 24 h later for 10min. Preference index was calculated as following.

(time to the new object - time to the familiar object)/total exploration time to the objects

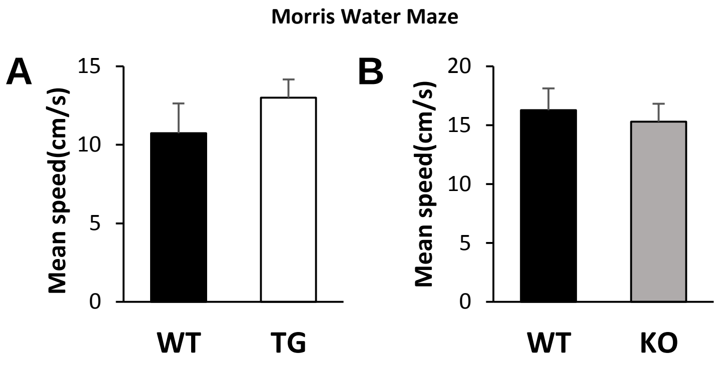

Figure S1. TREM2 does not affect basal locomotion. (A) Bar graphs calculated from the speed of TG and WT mice in Morris water maze test. **(B)** Bar graphs calculated from those of KO and WT mice. Data are presented as mean±SEM. One-way analysis of variance by Tukey's post-hoc comparisons tests was performed in all statistical analysis.

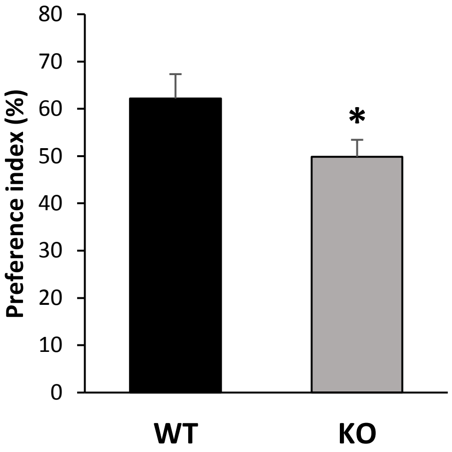

Figure S2. Novel object recognition was impaired in TREM2 KO mice. TREM2 KO mice display reduced novel object recognition compared with WT mice (WT, n=6 mice; TREM2 KO, n=7 mice). Data are presented as the mean ± SEM. (*$p < 0.05$, Student's t test).

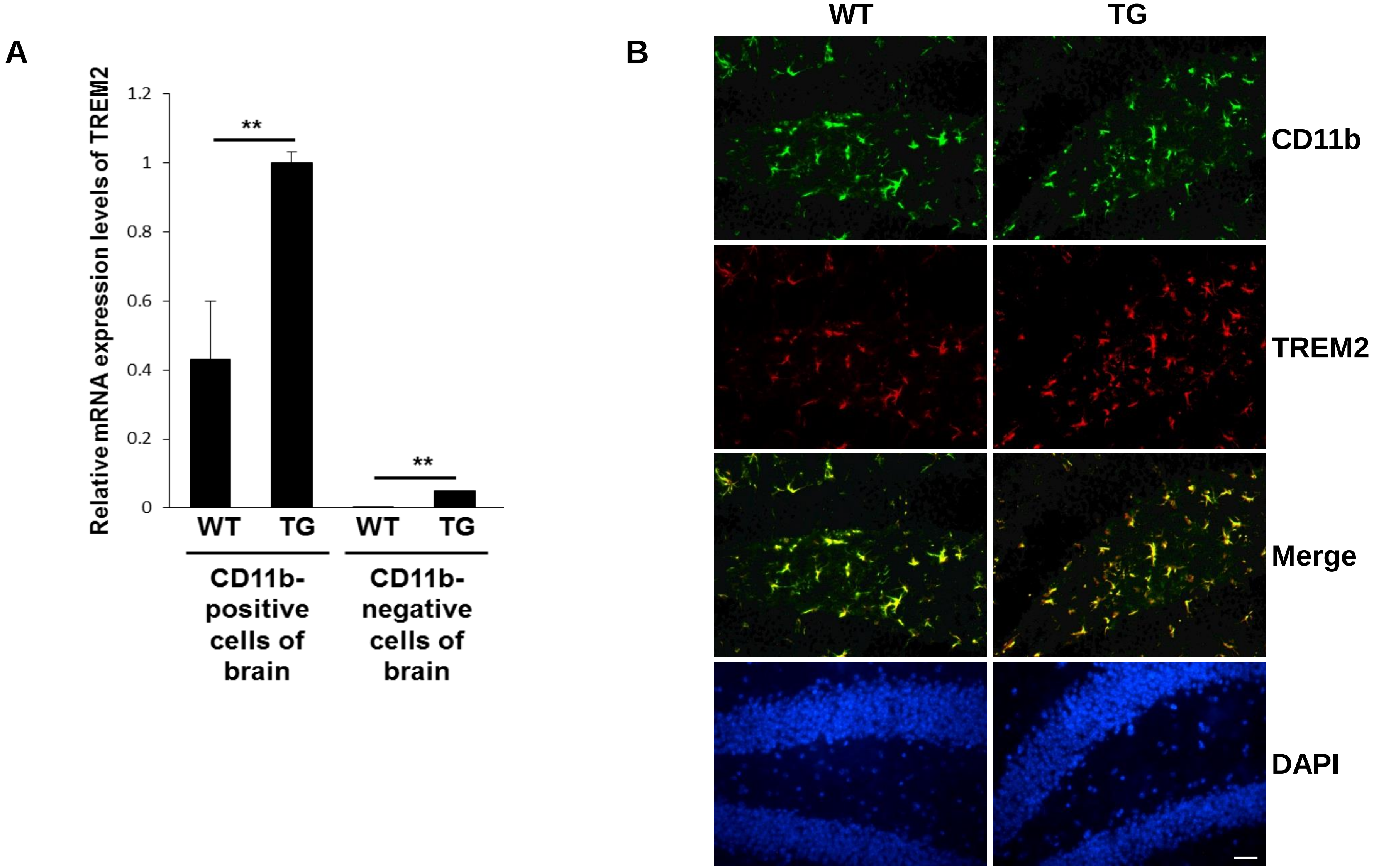
A
Relative mRNA expression levels of TREM2
1.2
1
0.8
0.6
0.4
0.2
0
**
**
WT
TG
WT
TG
CD11b-positive cells of brain
CD11b-negative cells of brain
B
WT
TG
CD11b
TREM2
Merge
DAPI

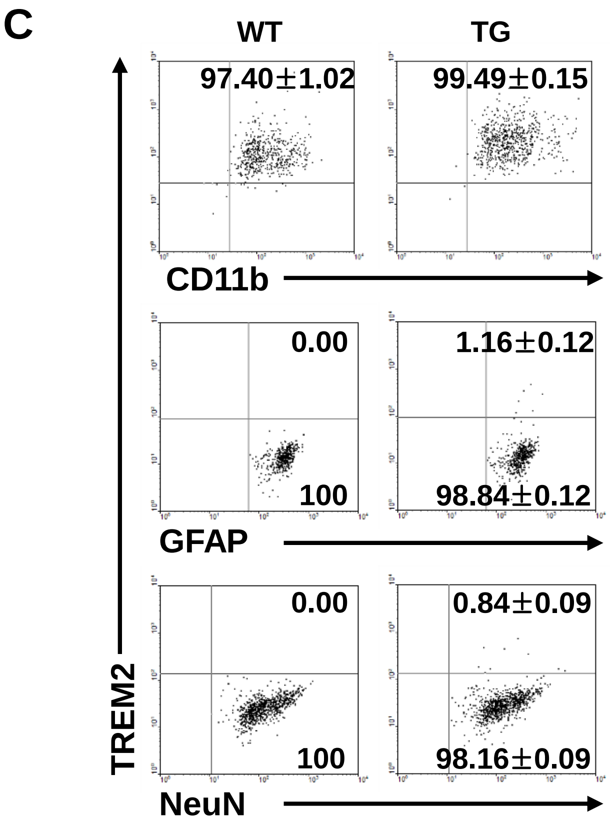

Figure S3. TREM2 expression was ubiquitously increased in TG mice with much higher level in microglia than in the other cell types. (A) TREM2 mRNA expression was determined in CD11b-positive and negative cells of the brain by real-time PCR. The cells were separated by MACS. (B) Brain slices were obtained from 1-year-old TG and WT mice, and the TREM2 expression pattern was evaluated by immunofluorescent staining using antibodies and DAPI as indicated. The stained brain slices were observed under fluorescence microscopy. Scale Bar: 50 µm. (C) Brain cells were isolated from 8-weeks-old TG and WT mice, and analyzed by flow cytometry using the indicated antibodies.

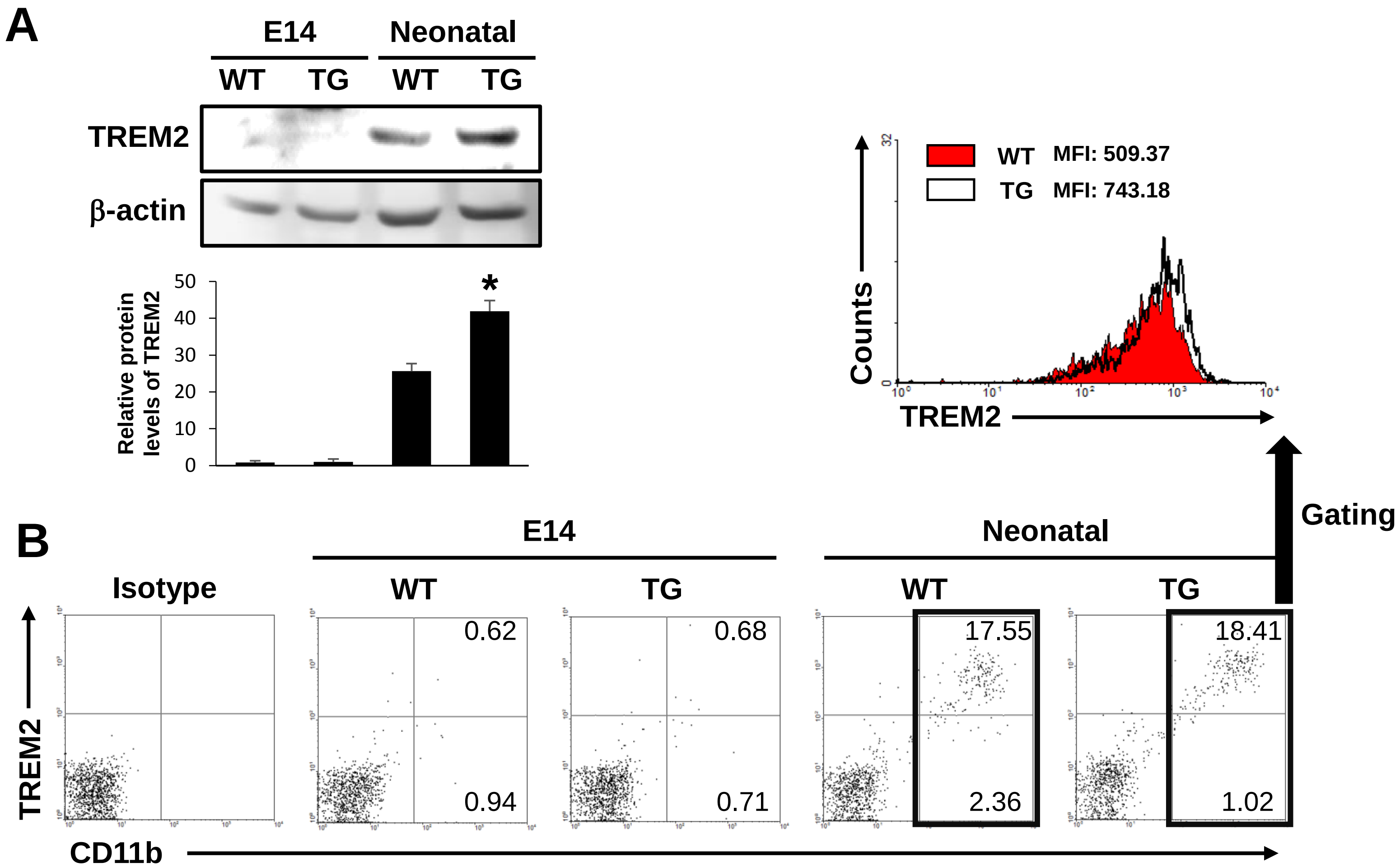

Figure S4. TREM2 expression levels in E14 and neonatal cultures from WT and TG mice. Cells were isolated from the brains of embryonic day 14 embryos (E14) or 2-day-old mouse pups (Neonatal). (A) TREM2 expression was assessed by western blot. After densitometric quantitation of the individual bands from western blot, the expression levels of TREM2 protein were calculated as relative TREM2 protein levels and are presented in a bar graph. (B) Cell surface expression of TREM2 was compared in an overlaying histogram after gating of the CD11b-positive population in a dot plot. The error bars represent the mean±SEM of three independent experiments (*$p < 0.05$).

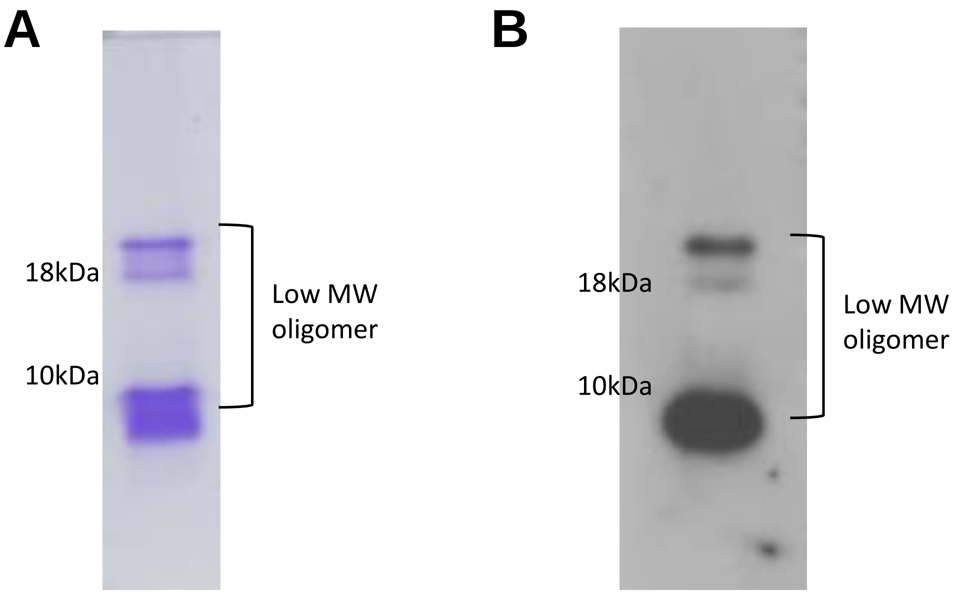

Figure S5. Production of Aβ oligomer. (A) The synthesized and oligomerized Aβ was detected by Coomassie Blue staining and (B) western blot using the antibody Ab5078p.

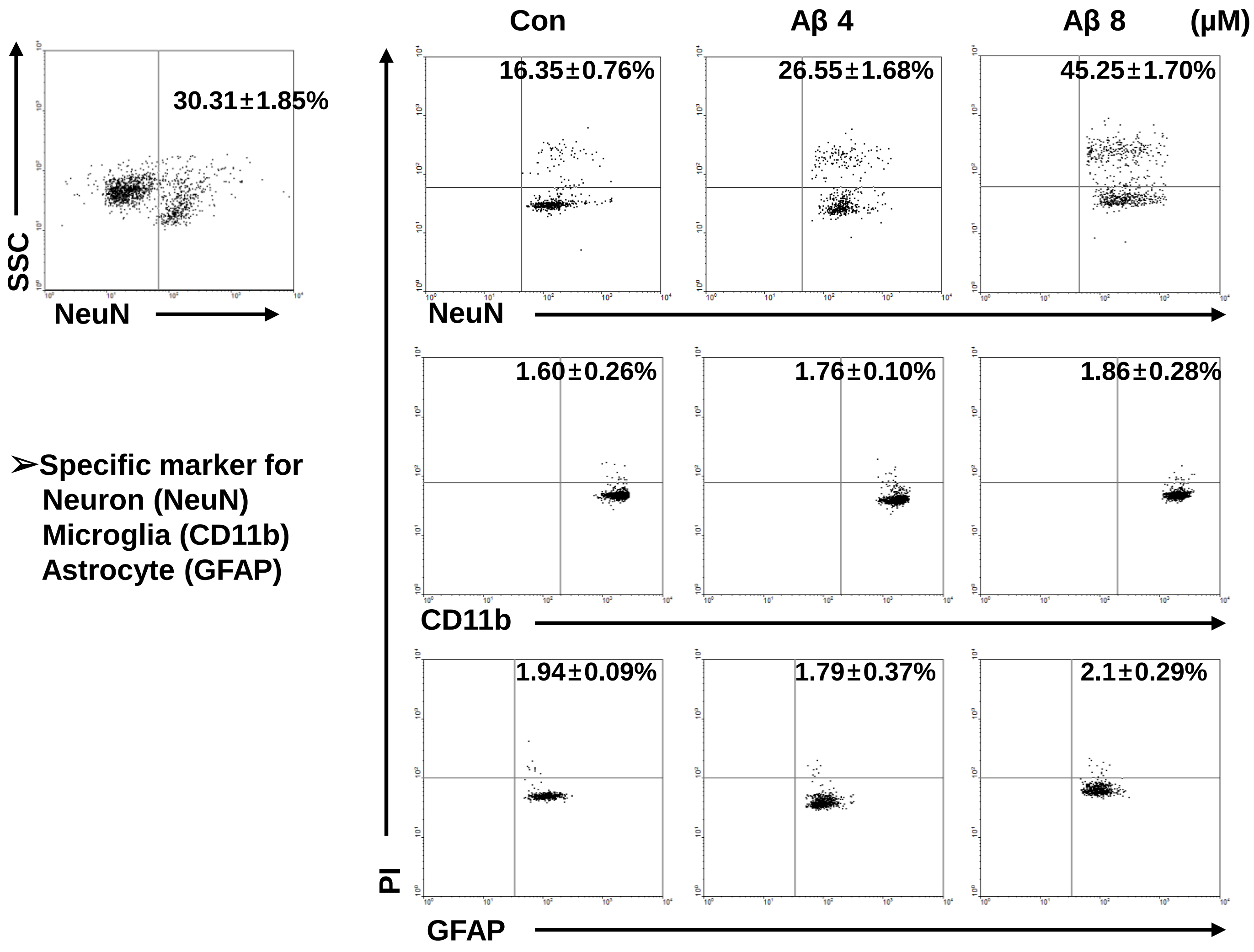

Figure S6. Identification of viable neuronal cells. After incubation of neonatal cultured cells of WT mice with Aβ for 3 days, the cells were stained with anti-NeuN (R&D), anti-GFAP (Dako A/S, Glostrup, Denmark), anti-CD11b and PI. The stained cells were analyzed by flow cytometry.

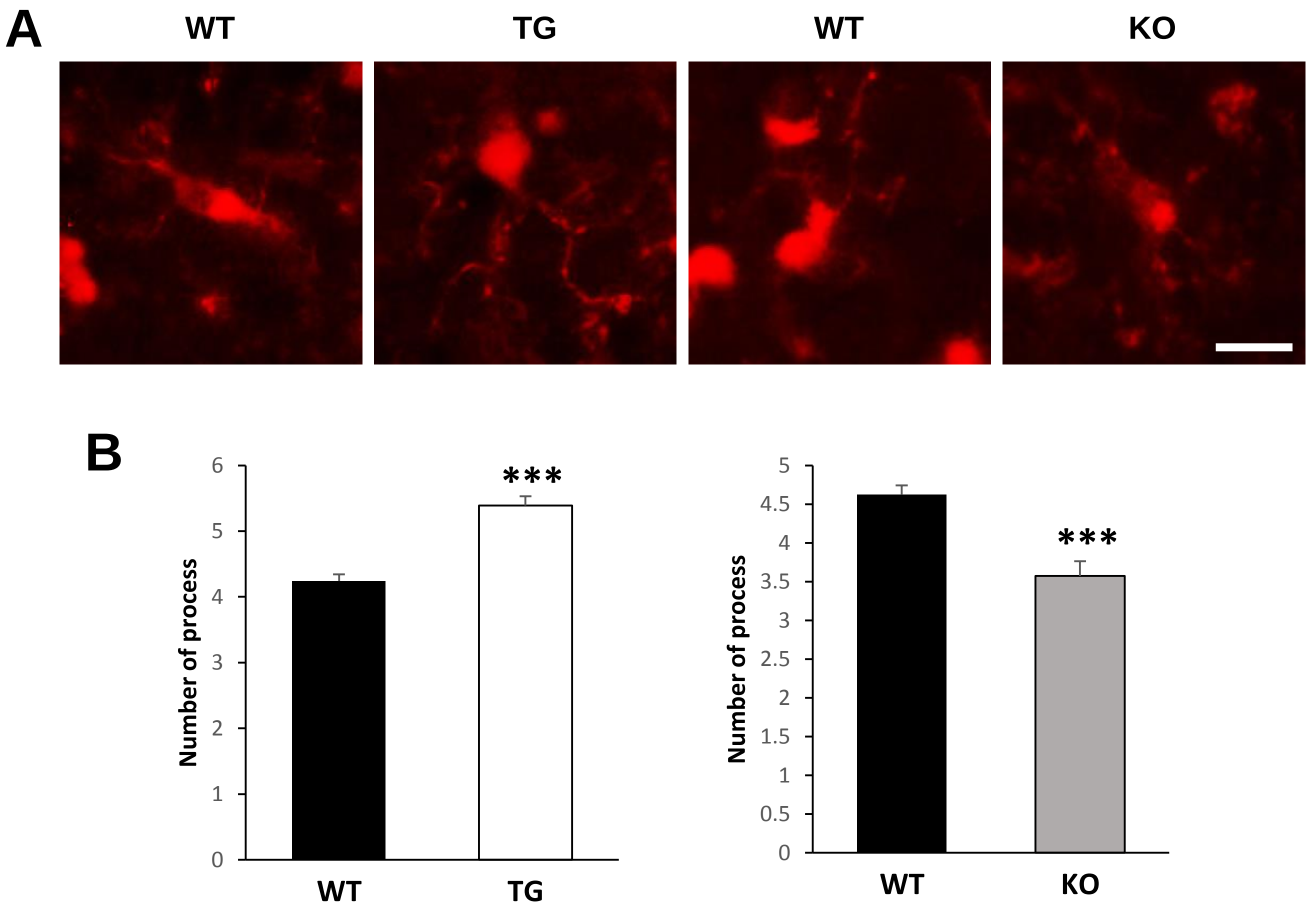

Figure S7. TREM2 regulated microglial activation in vivo. (A) Immunofluorescent staining using anti-Iba1 of TREM2 TG, KO and WT mice. Scale Bar: 50 μm (B) Average numbers of primary processes in individual Iba1-positive cells of 24-month-old mice of the indicated genotypes. Data are presented as the mean ± SEM. (***$p < 0.001$, Student's t test).

Figure S8

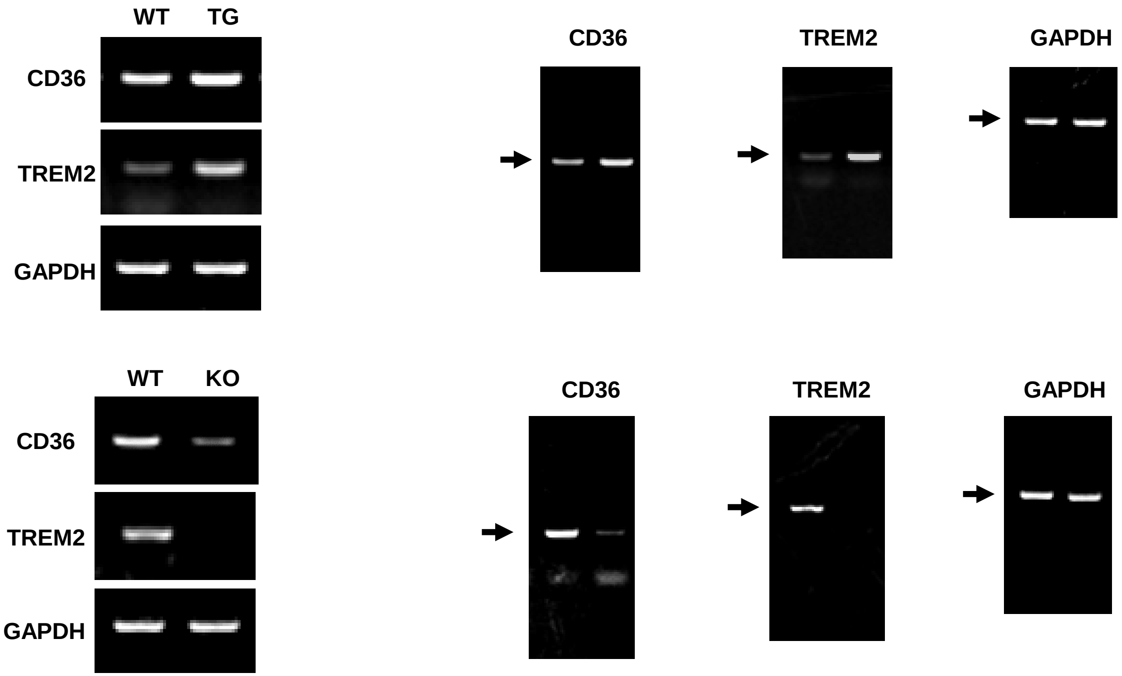

WT
TG
CD36
TREM2
GAPDH
CD36
TREM2
GAPDH
WT
KO
CD36
TREM2
GAPDH
CD36
TREM2
GAPDH

Figure S9

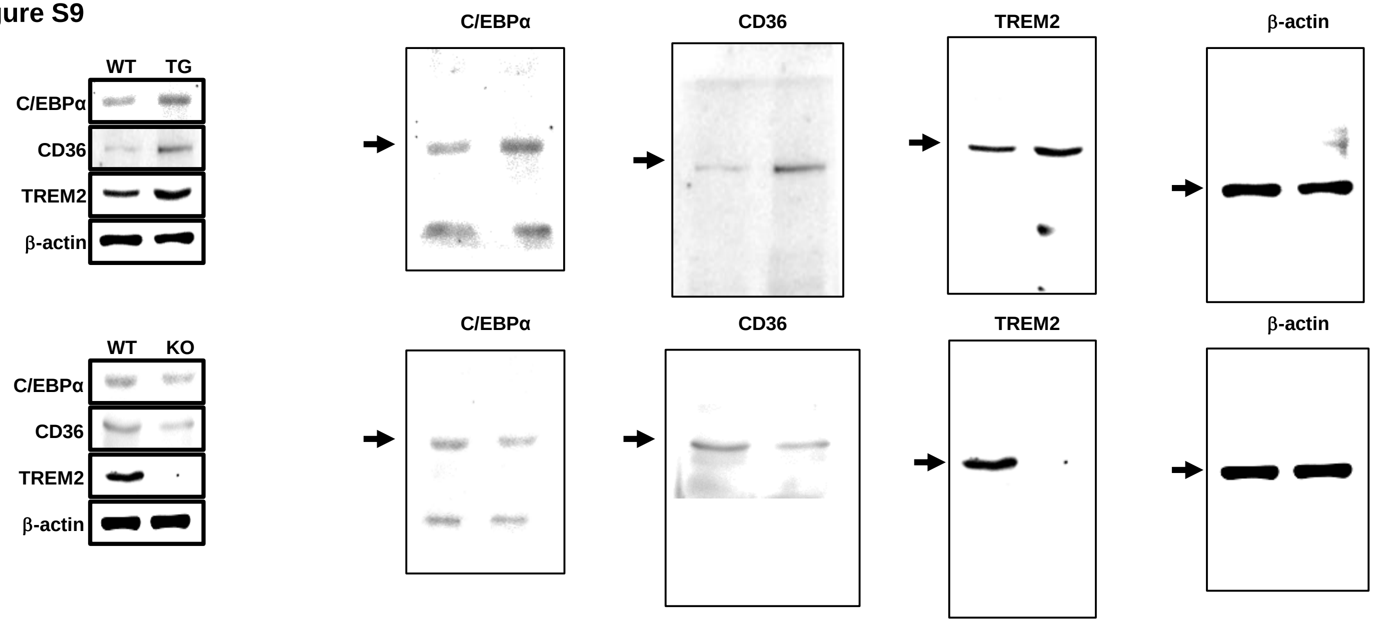

Figure S10

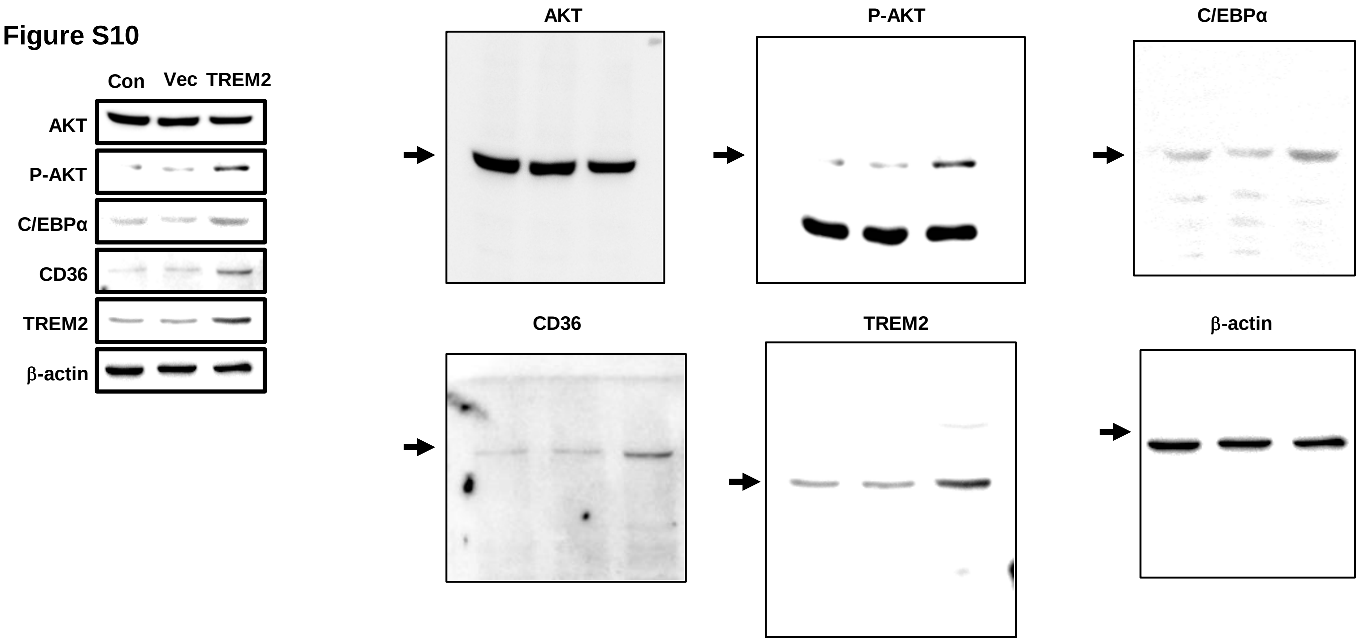

Figure S11

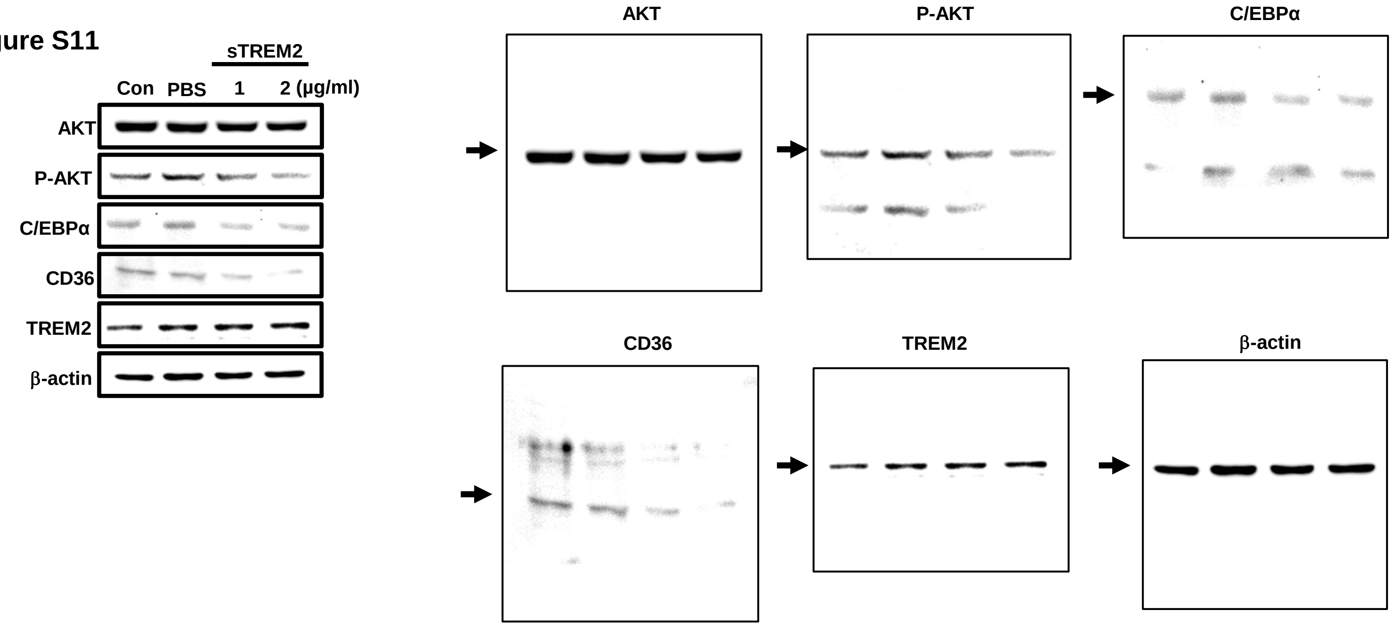

Figure S12

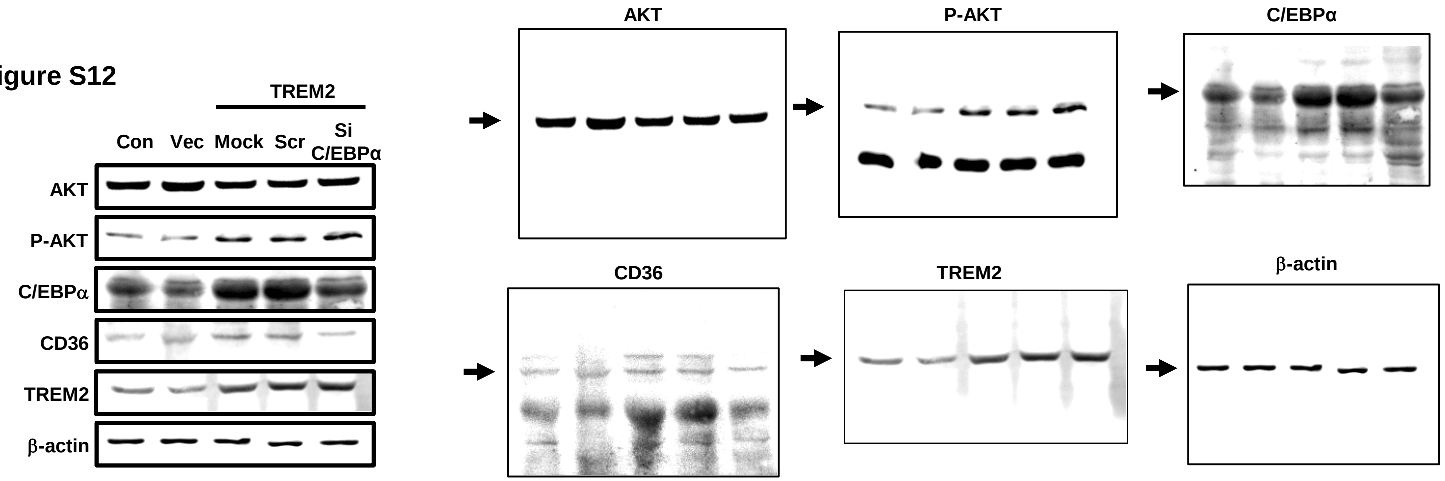

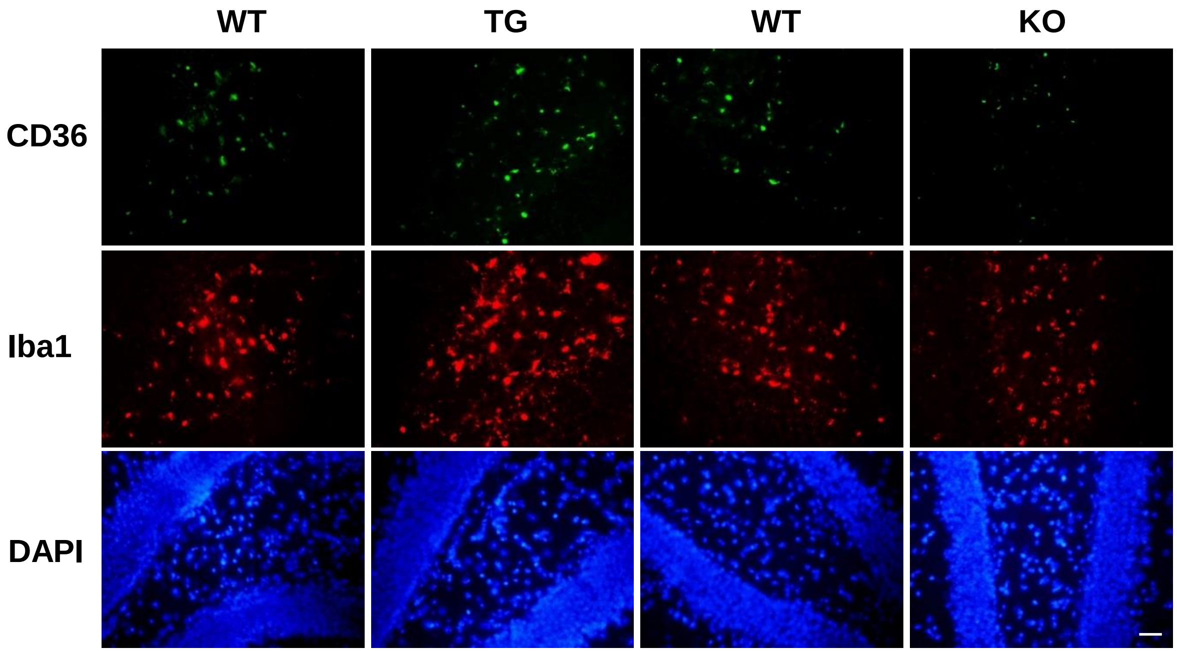

Figure S13. CD36 expression in the brains of TG and KO mice. Brain slices were obtained from 1-year-old TREM2 TG, KO, and WT mice, and immunofluorescent staining was performed using anti-TREM2, anti-Iba1 and DAPI. The stained brain slices were observed using fluorescence microscopy. Scale Bar: 50 μm.

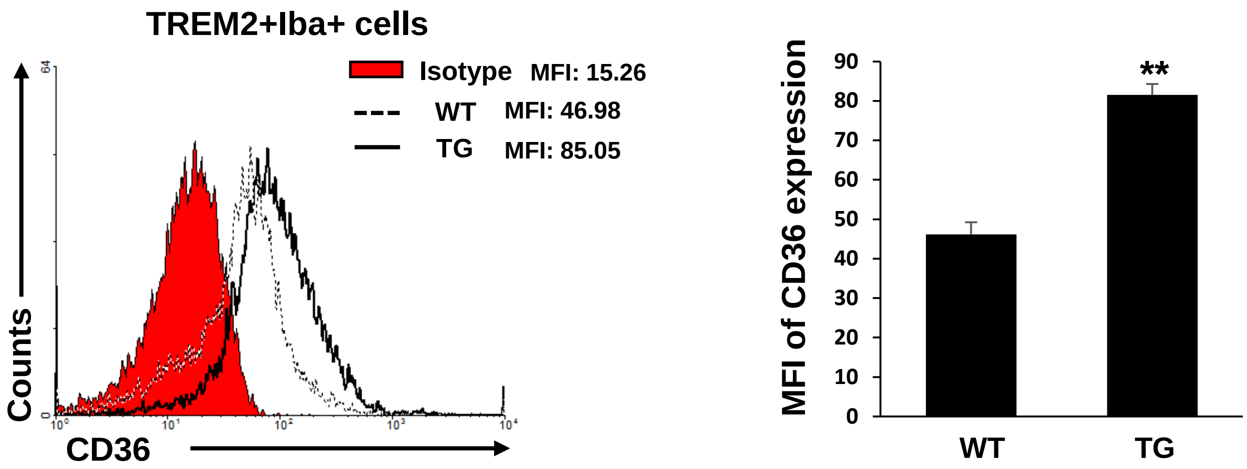

Figure S14. Higher CD36 expression in TREM2- and Iba1-positive cells of TG mice. . Brain cells were obtained from 8-weeks-old TREM2 TG and WT mice, and flow cytometry was performed using anti-TREM2, anti-Iba1 and anti-CD36 antibodies. The CD36 expression was analyzed in TREM2 and Iba1 double positive cells and presented by histogram (left). The MFI of CD36 were represented as a bar graph (right).
